# Analysis of the Guiding Role of CYP2C19 Gene Combined With Platelet Function Detection in Antiplatelet Therapy in Patients With Complex Coronary Artery Disease After PCI

**DOI:** 10.3389/fsurg.2022.839157

**Published:** 2022-02-09

**Authors:** Jiancai Yu, Yongxing Liu, Wanzhong Peng, Juan Liu, Ya Li, Junyan Liu, Yang Jiang, Demin Liu, Zesheng Xu

**Affiliations:** ^1^Department of Cardiology, Teaching Hospital of Cangzhou Central Hospital of Tianjin Medical University, Tianjin Medical University, Tianjin, China; ^2^Department of Cardiology, Cangzhou Central Hospital, Cangzhou, China

**Keywords:** CYP2C19, platelet function test, complex coronary artery lesions, PCI, antiplatelet therapy

## Abstract

**Objective:**

To explore the influence of CYP2C19 gene combined with platelet function test on clinical prognosis of patients with complex coronary artery disease receiving antiplatelet therapy after PCI.

**Methods:**

A total of 200 patients undergoing PCI in our hospital due to complex coronary artery disease from February 2019 to February 2021 were selected and divided into the control group and the observation group according to whether CYP2C19 gene detection was performed. The control group was treated with dual antiplatelet therapy of classical aspirin combined with clopidogrel, and the observation group was treated with individual antiplatelet therapy. The patients in the two groups were followed up for 1 year after PCI, and their quality of life was assessed using the Seattle Angina Questionnaire (SAQ score). The occurrence of major adverse cardiovascular events (MACE) during the follow-up period was also recorded.

**Results:**

The incidence of total MACE events in the observation group was slightly less than that in the control group, and the difference was statistically significant (*P* = 0.040). In particular, the observation group was superior to the control group in reducing the readmission rate of recurrent unstable angina pectoris, and the difference was statistically significant (*P* = 0.023). The location of coronary culprit lesions with recurrent ischemic events was commonly seen in non-interventional target lesions (interventional/non-interventional target sites: 12.9%: 77.1%). The SAQ score in the observation group was larger than that in the control group, and the difference was statistically significant (*P* = 0.012). There was no statistical difference in the incidence of major bleeding between the two groups (*P* = 0.352).

**Conclusion:**

Using CYP2C19 genotype combined with platelet function test to guide individualized antiplatelet therapy after complex coronary artery PCI is beneficial to reducing ischemic events in a short period (1 year), mainly due to reducing the risk of readmission for recurrent unstable angina pectoris, and improving the quality of daily life of patients without increasing the risk of massive hemorrhage, which can improve clinical prognosis.

## Introduction

Dual anti-platelet therapy (DAPT) composed of aspirin combined with a P2Y12 receptor inhibitor is the cornerstone for the prevention of cardiac and systemic ischemic events in patients with coronary heart disease (1, 2). It is recommended by a large number of clinical studies and domestic and foreign guidelines that DAPT should be accepted after coronary artery percutaneous coronary intervention (PCI). With the recent iterations of drug-eluting stents, the promotion of potent P2Y12 receptor inhibitors, and the continuous updating of other combined drug strategies, DAPT has many new options compared with classical regimens in terms of drug selection, timing, and optimal duration (3, 4). Antiplatelet drug therapy differs among individuals based on genetic differences and individual factors, which are significantly associated with the occurrence of adverse events such as ischemia or hemorrhage. Clinicians expect an appropriate approach to guide antiplatelet therapy after PCI to improve the clinical outcome of coronary heart disease. With the development of clinical trials in recent years, CYP2C19 gene and platelet function testing have become a hot topic to guide DAPT treatment after PCI, and more clinical evidence has been accumulated (5, 6). Although the recently released Chinese Experts Consensus on Dual-antiplatelet Therapy for Coronary Heart Disease points out that routine platelet function and genotyping tests are not supported based on current evidence to guide the selection of antiplatelet strategies, they can be applied in specific cases. For example, patients with high ischemic risk factors can receive DAPT escalation therapy with gene detection and platelet function guidance, and patients with high bleeding risk can receive DAPT escalation therapy (7, 8). The enrolled populations in this study were patients with complex coronary artery lesions (including CTO lesions, calcified lesions, left main artery lesion, bifurcated lesions, three-vessel lesion, lesions with the total stent length >60 mm and more than three stents implanted in a single time), which were high risk factors for ischemia and had high risk of ischemic adverse cardiovascular events. According to the statistics of previous studies, the incidence of MACE events in patients with complex coronary lesions within 1 year after PCI is as high as 10.4–19.7%, which seriously affects the clinical outcome (9, 10). Therefore, in this study, CYP2C19 gene combined with platelet function testing was intended to guide individualized antiplatelet therapy for patients with complex coronary artery lesions after PCI, and to explore its impact on clinical prognosis.

## Materials and Methods

### Patients

A total of 200 patients who were confirmed with complex coronary artery lesions by coronary angiography and successfully completed PCI in Cangzhou Central Hospital from February 2019 to February 2021 were selected as the research subjects. This study was approved by the Ethics Committee of our hospital and has completed the national clinical study registration. It was conducted with the informed consent of the patients and met the following inclusion criteria: Meet the diagnostic criteria for coronary heart disease and meet the criteria for PCI confirmed by coronary angiography and/or intravascular ultrasound (IVUS) (11, 12); Patients with complex coronary artery lesions, including coronary CTO lesions, calcified lesions, left main artery lesion, bifurcated lesion, three-vessel lesion, lesions with the total stent length >60 mm, and lesions requiring more than three stents implantation at a time. Exclusion criteria: Patients who used P2Y12 receptor antagonist within 30 days before operation; Patients who had hemorrhagic stroke in the past, ischemic stroke or cerebrovascular event within 6 months, and rational bleeding due to active diseases (such as peptic ulcer or upper gastrointestinal bleeding); Patients with contraindications to use tigrew or clopidogrel or drug allergy; Patients with malignant tumor and expected survival time of not more than 1 year; Patients with moderate to severe liver and kidney damage and coagulation dysfunction; Patients who are going to undergo CABG in the near future; Patients who failed to cooperate with the completion of the experiment.

### Research Methods

#### Method of Administration

The patient was given a load of 300 mg of clopidogrel (75 mg^*^7 tablets/box, Sanofi, France) and aspirin (100 mg^*^30 tablets/box, Bayern Chemie) 30 min before PCI, and a maintenance dose of clopidogrel 75 mg/d + aspirin 100 mg/d from the next day. Patients with elective interventional therapy were given clopidogrel 75 mg/d + aspirin 100 mg/d for at least 4 days.

#### Surgical Methods

The operation was completed using Siemens cardiovascular imaging machine. The operator had mature experience in PCI of complicated coronary arteries, and more than half of the cases were completed under the guidance of intravascular ultrasound.

#### Grouping and Administration

Fasting blood was collected in the morning of the first day after PCI for CYP2C19 gene polymorphism detection, and sent to our central laboratory. The results were reported within 6 h. They were divided into the control group (*n* = 100) and the observation group (*n* = 100) according to whether or not CYP2C19 gene testing was performed. Patients in the control group after PCI continued to take aspirin combined with clopidogrel antiplatelet regimen (aspirin 100 mg, once a day; Clopidogrel 75 mg, once a day). The observation group according to the CYP2C19 genotype will be divided into three groups of patients with rapid metabolism (ultra-fast metabolism and fast metabolism), intermediate metabolism and slow metabolism. Patients with rapid metabolism continued to take aspirin and clopidogrel orally; Patients with slow metabolism were adjusted for aspirin in combination with tigroid DAPT regimen (aspirin 100 mg, once a day; ticagrelor 90 mg, twice a day); Platelet function of patients in the intermediate metabolic group was tested 5 days after surgery, and patients in this group were subdivided into clopidogrel normal response group (NCR; Maximal platelet aggregation rate <46) and clopidogrel low response type group (LCR; Maximum platelet aggregation rate ≥46). Patients in the NCR group continued to take aspirin in combination with clopidogrel. Patients in the LCR group were adjusted to aspirin combined with ticagrelor antiplatelet therapy.

### Observation Index

Major adverse cardiovascular events (MACE) include unstable angina, recurrent non-fatal acute myocardial infarction, stent thrombosis, unexpected revascularization of rake vessels, cardiac death, ischemic stroke, and massive hemorrhage. Cardiac death is defined as death from any definite cardiovascular cause. Stent thrombosis is defined as the formation of thrombus at the original stent implantation caused by any cause after PCI, which leads to complete or incomplete coronary artery obstruction. Ischemic stroke is defined as transient ischemic attack, cerebral infarction, etc. Massive hemorrhage was defined as BRAC standard type 3 (13). Quality of life was evaluated based on Seattle Angina Questionnaire (SAQ) (14): It was divided into five items and 19 items, including: degree of physical activity limitation (Question 1), stable angina pectoris (Question 2), attack of angina pectoris (Questions 3–4), treatment satisfaction (Questions 5–8), and disease cognition (Questions 9–11). The 19 items in the five items were scored item by item as well as the SAQ total score, which was then converted into a standard score according to the formula. The standard score = (actual score—the lowest score in this aspect)/(the highest score in this aspect—the lowest score in this recipe) × 100. The higher the score was, the better the quality of life and body functional status would be.

### Clinical Data Collection

Detailed clinical data of the patients were collected: (1) General data: gender, age, weight, BMI, smoking history and drinking history. (2) Previous medical history: hypertension, diabetes, cerebral infarction and coronary heart disease. (3) Blood tests: white blood cell count (WBC), hemoglobin (HGB), platelet count (PLT), alanine aminotransferase (ALT), aspartate aminotransferase (AST), creatinine (CR), uric acid (UA), glycated hemoglobin, triglycerides (TG), total cholesterol (TC), high density lipoprotein (HDL-L), low density lipoprotein (LDL-C). (4) Cardiac function: left ventricular diastolic diameter and EF value. (5) Clinical medication: angiotensin converting enzyme inhibitor (ACEI)/angiotension receptor blocker (ARB), β -receptor blockers, statins, calcium-channel antagonist (CCB), proton pump inhibitor (PPI). (6) Surgical conditions: IVUS utilization rate and immediate success rate of surgery (residual stenosis of the lesion <10% and TIMI blood flow grade 3).

### Follow-Up

All patients included in the present study were followed up for 9~12 months after PCI through the department follow-up system, telephone, readmission tracking, and so on. If the patients were not contacted for more than three times through the appeal method, they were considered lost to follow-up (9). A total of 200 patients were included in this study. A total of 195 cases were successfully followed up, including 97 cases in the control group and 98 cases in the observation group.

### Statistical Methods

The results of this experiment were statistically analyzed by SPSS 20.0 (SPSS Co., Ltd., Chicago, USA). Count data were expressed by (rate), and chi-square test was used for their comparison between groups. Measurement data were expressed by (mean ± standard deviation), and *t*-test was used for their comparison between groups. *P* < 0.05 indicates that the difference is statistically significant.

## Results

### Comparison of Baseline Information

There was no statistically significant difference between the two groups in gender, age and other baseline data (*P* > 0.05). As shown in Table 1.

**Table 1:** Comparison of baseline data between the two groups.

**Baseline information**	**Control group (***n*** = 97)**	**Observation group (***n*** = 98)**	***t*** **/χ^2^**	***P***
Age (years)	68.40 ± 9.10	67.38 ± 8.50	0.808	0.417
Gender [male (%)]	54 (55.67)	46 (46.94)	1.487	0.253
Body weight (kg)	70.23 ± 9.28	68.12 ± 9.45	1.573	0.118
BMI (kg/m^2^)	24.99 ± 1.97	24.84 ± 2.17	0.505	0.614
Smoking history [*n* (%)]	32 (32.99)	31 (31.63)	0.041	0.879
Drinking history [*n* (%)]	22 (22.68)	19 (19.39)	0.318	0.602
**Past medical history [*****n*** **(%)]**				
Hypertension	24 (24.74)	29 (29.59)	0.579	0.520
Type 2 diabetes	26 (26.80)	24 (24.49)	0.137	0.745
Cerebral Infarction	14 (14.43)	17 (17.35)	0.309	0.696
Coronary heart disease	26 (26.80)	28 (28.57)	0.046	0.873
**Blood examination**				
$\text{WBC}(10\hat{9}\text{L})$	7.45 ± 1.61	7.34 ± 1.53	0.489	0.623
$\text{HGB}(10\hat{9}\text{L})$	129.82 ± 10.54	131.15 ± 13.88	0.753	0.453
$\text{PLT}(10\hat{9}\text{L})$	206.56 ± 62.92	212.55 ± 63.13	0.664	0.508
ALT (U/L)	31.64 ± 6.74	32.44 ± 9.24	0.690	0.491
AST (U/L)	32.73 ± 8.46	30.96 ± 8.37	1.469	0.143
CR (μmol/L)	91.32 ± 15.73	87.21 ± 16.70	1.769	0.079
UA (μmol/L)	355.55 ± 70.74	368.94 ± 88.33	1.168	0.244
HbA1c (%)	5.75 ± 0.79	5.77 ± 0.68	0.189	0.843
TC (μmol/L)	4.68 ± 0.55	4.81 ± 0.44	1.854	0.056
TG (μmol/L)	1.78 ± 0.43	1.80 ± 0.27	0.538	0.550
HDL-L (μmol/L)	1.27 ± 0.39	1.17 ± 0.23	2.709	0.027
LDL-C (μmol/L)	3.58 ± 0.31	3.65 ± 0.43	1.303	0.202
**Cardiac function**				
Left ventricular diastolic diameter (mm)	48.26 ± 4.61	49.08 ± 4.74	1.224	0.220
EF (%)	54.46 ± 4.80	53.87 ± 4.03	0.929	0.354
**Clinical medication [*****n*** **(%)]**				
ACEI/ARB	87 (89.69)	89 (90.82)	0.071	0.814
Statin	88 (90.72)	91 (92.86)	0.295	0.624
β-blocker	87 (89.69)	90 (91.84)	0.268	0.630
CCB	10 (10.31)	7 (7.14)	0.614	0.459
PPI	95 (97.94)	92 (93.88)	2.043	0.153
**Surgery [*****n*** **(%)]**				
IVUS use	46 (47.42)	43 (43.88)	0.658	0.248
PCI immediate success	92 (94.85)	94 (95.92)	0.128	0.721

### Grouping and Distribution of CYP2C19 Genotype in the Observation Group

A CYP2C19 genotype subgroup (98 cases) involving only observation group patients was conducted and the subgroup met the 2013 CPIC guidelines. The fast metabolism group included 33 patients (33.68%) with fast metabolism progressive CYP2C19^*^1/^*^1 and ultrafast metabolism progressive CYP2C19^*^1/^*^17. The intermediate metabolic group included patients with CYP2C19^*^1/^*^3, CYP2C19^*^1/^*^2, CYP2C19^*^2/^*^17, and CYP2C19^*^3/^*^17 (36 cases, or 36.73%); The slow metabolic group included patients with CYP2C19^*^2/^*^2, CYP2C19^*^3/^*^3, and CYP2C19^*^2/^*^3 (29 cases, or 29.59%). As shown in Table 2.

**Table 2:** Grouping and distribution of CYP2C19 genotype in the observation group.

**Metabolic type**	**Genotype**	**Cases (*n*)**	**Percentage (%)**
Fast metabolism type (*n* = 32)	*1/*1	19	19.39
	*1/*17	14	14.29
Intermediate metabolic (*n* = 30)	*1/*3	9	9.18
	*1/*2	10	10.21
	*2/*17	9	9.18
	*3/*17	8	8.16
Slow metabolism type (*n* = 36)	*2/*2	11	11.22
	*3/*3	10	10.21
	*2/*3	8	8.16

According to gene distribution, the gene frequencies at ^*^2, ^*^3, and ^*^17 loci, the actual genotype frequency and the theoretical genotype frequency were counted, and the analysis of variance test was used to test the actual genotype frequency and the theoretical genotype frequency at ^*^2, ^*^3, and ^*^17 loci. The results showed that there was no significant difference between the actual genotype frequency and the theoretical genotype frequency (P > 0.05), which was in line with the Hardy–Weinberg genetic balance test, indicating that the gene frequency of the sample population was in line with the genetic balance rule and was representative.

### Comparison of Incidence of MACE Events

**Figure 1:**
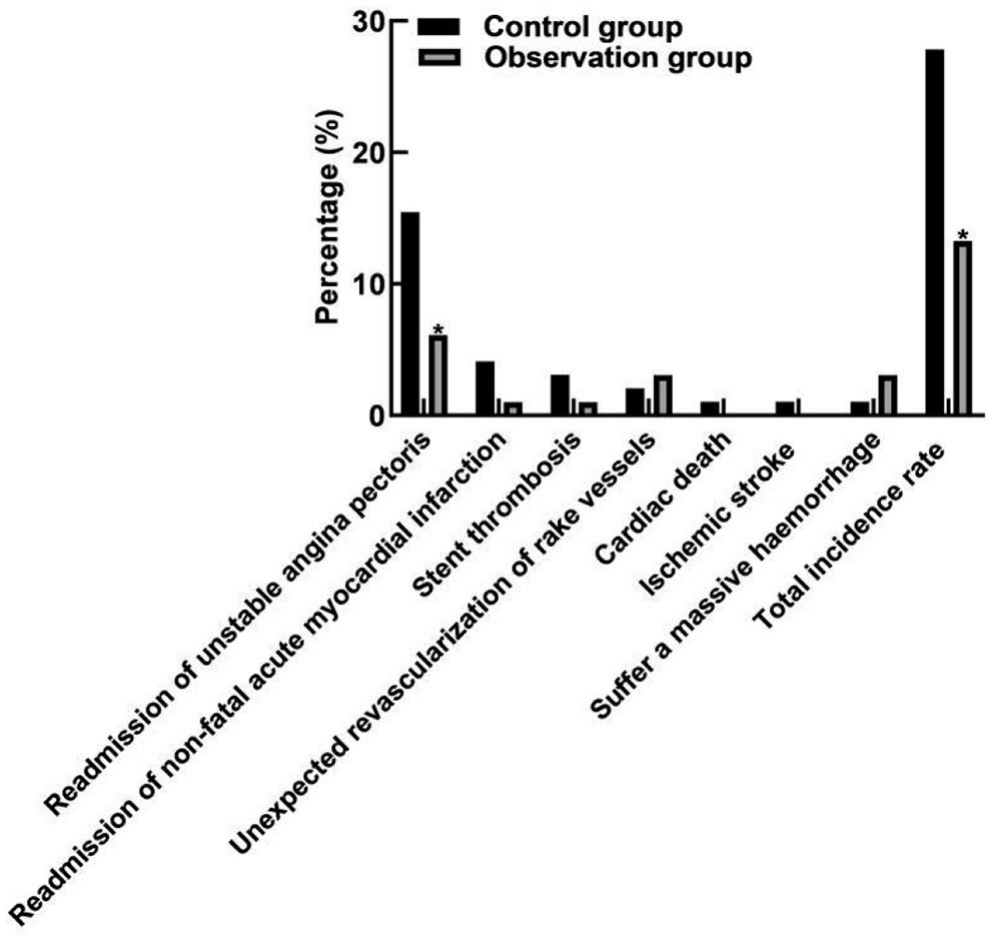
Comparison of MACE event incidence between two groups. Compared with the control group, *P < 0.05.

Comparison of MACE event results between the two groups showed that although the individualized antiplatelet treatment group (observation group) did not show statistical difference in ischemic adverse events such as re-admission of non-fatal acute myocardial infarction, stent thrombosis, unexpected revascularization of rake vessels, cardiac death, and ischemic stroke (*P* > 0.05), it benefited from reducing the risk of re-admission for recurrent unstable angina pectoris (*P* = 0.040). The total incidence of MACE events in the observation group was less than that in the control group, and the difference was statistically significant (*P* = 0.046). Moreover, the incidence of massive hemorrhage did not increase (*P* = 0.352). As shown in Figure 1.

### Comparison of SAQ Scores Between the Two Groups

**Figure 2:**
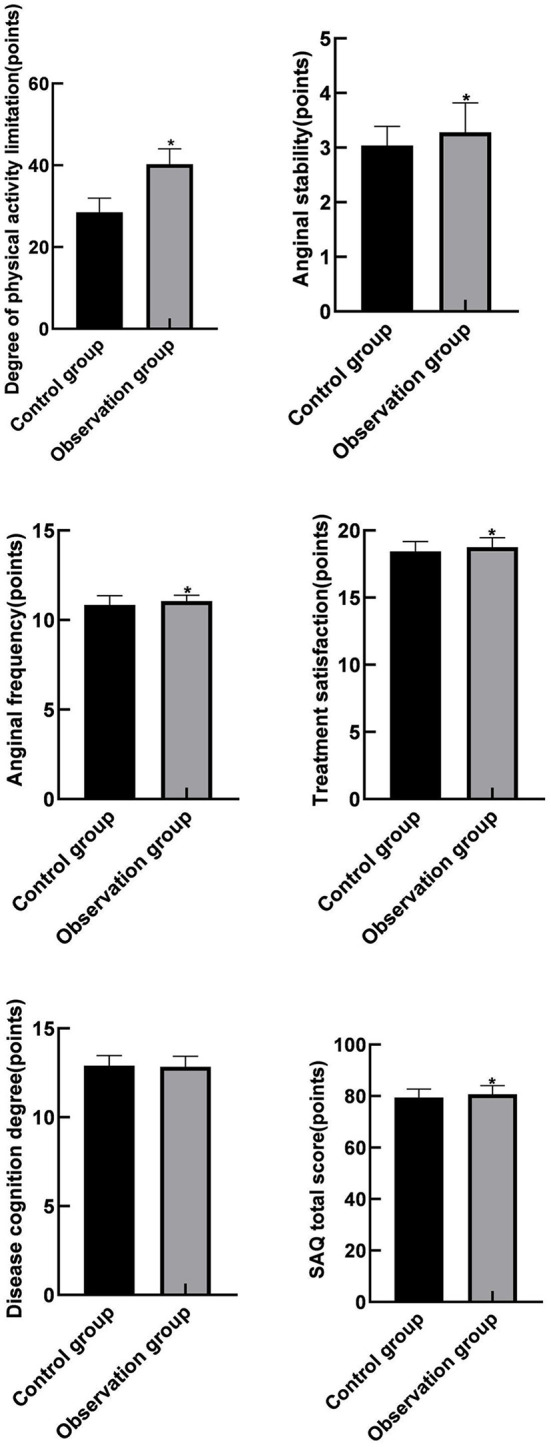
Comparison of SAQ scores between the two groups. Compared with the control group, *P < 0.05.

In view of the fact that the observation group could reduce the readmission risk of recurrent unstable angina pectoris, the Seattle Angina Survey Scale was used in this experiment to analyze the quality of life of patients in two groups. Among them, the observation group was significantly superior to the control group in the aspects of limited physical activity, stable state of angina, onset of angina and treatment satisfaction, with a statistical difference (*P* < 0.05). The difference was basically the same in the disease cognition, with no statistical difference (*P* = 0.062). As shown in Figure 2.

### Analysis of the Location of Offenders With Recurrent Ischemic Events in the MACE Event

Further, we wanted to determine the lesion location of criminals who excluded stent thrombosis and died from cardiac causes in the MACE event and experienced another ischemic event. As a result, CAG was reexamined in a total of 31 patients who were readmitted for recurrent unstable angina pectoris and non-fatal acute myocardial infarction and who underwent unexpected revascularization of target vessels. Among them, 4 cases (12.90%) were located at interventional target lesion, and 27 cases (87.10%) were located at non-interventional target lesion. Hence, non-interventional target lesion sites were more common.

## Discussion

Aspirin combined with P2Y12 receptor inhibitors is the basis of antiplatelet therapy after PCI. However, individual platelet drug therapy shows diversity differences, which are significantly associated with the occurrence of adverse events such as recurrent thrombosis or hemorrhage (15, 16). It has been found in clinical practice that patients after complicated coronary artery PCI are more likely to suffer from such adverse cardiovascular events as angina pectoris, revascularization of target vessels, readmission for non-fatal myocardial infarction, and cardiogenic death. Even after standardized DAPT treatment, the probability of such adverse cardiovascular events is still significantly higher than that of common lesions (17, 18). The reason was related to clopidogrel resistance and low clopidogrel response in addition to surgical factors. Clopyrrole resistance is related to a variety of factors, of which genetic factors play a significant role (19, 20). Previous studies have found that Asian patients have a greater risk of adverse cardiovascular events than western populations, which may be related to the high proportion of CYP2C19 gene variation in Asian (21). However, the application of clopidogrel gene detection alone to distinguish the population with clopidogrel metabolism cannot independently predict the occurrence of cardiovascular ischemic events. It was also found in the clinical practice that ischemic adverse events after PCI were associated with platelet reactivity (22). Because studies have found that even after the application of CYP2C19 gene testing guidance and the administration of standard DAPT drug dose treatment, 20–30% of patients with coronary heart disease still have high platelet reactivity (HPR), and about 5–6% of patients after stent implantation have DAPT resistance, which is more likely to occur in patients in East Asia (23, 24). Based on this, clinicians expect to find an appropriate method to guide antiplatelet therapy after PCI, so as to capture the pros and cons of the triggered endpoint events and make them become the method to optimize and guide antiplatelet therapy after PCI, so as to improve the clinical outcome of coronary heart disease. Unfortunately, no consensus has been reached in the worldwide regarding the optimal guidance of antiplatelet drug therapy after PCI.

The purpose of this experiment is to initially explore whether CYP2C19 gene combined with platelet function testing can improve the clinical prognosis of individualized antiplatelet therapy after PCI, as compared with the classical aspirin combined with clopidogrel DAPT regimen. Therefore, our subgroup design was not complex, and although the observation group involved multiple subgroups, we were only concerned with the overall clinical outcome. As a result, the individualized antiplatelet therapy group (observation group) showed no statistical difference in ischemic adverse events such as readmission of non-fatal acute myocardial infarction, stent thrombosis, unexpected revascularization rake vessels, cardiac death, and ischemic stroke. The possible reason is that the ischemic events in this part may be more related to the surgical factors or to the stability of the interventional target lesion. In recent years, with the innovation of interventional instruments, endovascular examination methods and the improvement surgical techniques, ischemic events (especially in-stent thrombosis and restenosis) after PCI have been significantly reduced, so there is not enough room to change the clinical outcome through antiplatelet drug therapy. In this study, about half of the PCI operations were conducted under the guidance of IVUS. Moreover, the surgeons had rich experience in complex coronary intervention, and the immediate success rates of the operations were above 90%, which could basically eliminate the surgical bias.

Different from other similar studies, the results of this experiment showed that the individualized antiplatelet therapy could reduce the readmission risk of recurrent unstable angina pectoris and improve the quality of daily life of patients, which might be related to the promotion of tigroid therapy, which could more effectively inhibit platelet aggregation, improve endothelial function, reduce plaque erosion and rupture, and provide better blood flow reserve. This part of the effect might be more on non-interventional target locations, reducing ischemic events caused by plaques outside of the interventional target locations. Therefore, we further analyzed the location of the lesions in the MACE events that excluded patients with stent thrombosis and cardiogenic death, and recurred ischemic events The results confirmed that the lesion sites of recurrent ischemic events were mainly located in non-intervention target lesions, and the promotion to tigroid therapy improved the stability of non-intervention target lesions and reduced the ischemic events caused by the location of non-intervention target lesions.

## Conclusion

Using CYP2C19 genotype combined with platelet function test to guide individualized antiplatelet therapy after complex coronary artery PCI is beneficial to reducing ischemic events in a short period (1 year), mainly due to reducing the risk of readmission for recurrent unstable angina pectoris, and improving the quality of daily life of patients without increasing the risk of massive hemorrhage, which can improve clinical prognosis. Of course, when choosing antiplatelet drugs in clinical practice, patients' risk of ischemia and hemorrhage should be considered comprehensively, which is sometimes difficult to grasp. At this time, gene and platelet detection are considered as optional tools to guide treatment.

## Data Availability Statement

The original contributions presented in the study are included in the article/supplementary material, further inquiries can be directed to the corresponding author/s.

## Ethics Statement

The studies involving human participants were reviewed and approved by Ethics Committee of Cangzhou Central Hospital (2019002). The patients/participants provided their written informed consent to participate in this study.

## Author Contributions

JY is mainly responsible for the writing of the paper. YoL and WP are mainly responsible for the design of the research. JuaL and YaL are mainly responsible for the detection and evaluation of the results. JunL is mainly responsible for the recording of the research results. DL is mainly responsible for the statistics of the result data. ZX is mainly responsible for the guidance of the entire research. All authors contributed to the article and approved the submitted version.

## Funding

This study was funded by the Medical Science Research Project of Hebei Province (20200176).

## Conflict of Interest

The authors declare that the research was conducted in the absence of any commercial or financial relationships that could be construed as a potential conflict of interest.

## Publisher's Note

All claims expressed in this article are solely those of the authors and do not necessarily represent those of their affiliated organizations, or those of the publisher, the editors and the reviewers. Any product that may be evaluated in this article, or claim that may be made by its manufacturer, is not guaranteed or endorsed by the publisher.
